# Thyroidectomy and Its Complications: A Comprehensive Analysis

**DOI:** 10.3390/biomedicines13020433

**Published:** 2025-02-11

**Authors:** Ignazio Gerardi, Barbara Verro, Roberta Amodei, Pierina Richiusa, Carmelo Saraniti

**Affiliations:** 1 Section of Otolaryngology, Department of Biomedicine, Neuroscience and Advanced Diagnostics, BIND, University of Palermo, 90127 Palermo, Italy; ignazio.gerardi@gmail.com (I.G.); carmelo.saraniti@unipa.it (C.S.); 2 Affiliation Section of Endocrinology and Diabetology, Health Promotion, Department of Health Promotion, Mother and Child Care, Internal Medicine and Medical Specialties “G. D’Alessandro”, PROMISE, University of Palermo, 90127 Palermo, Italy; roberta.amodei@gmail.com (R.A.); pierina.richiusa@policlinico.pa.it (P.R.)

**Keywords:** thyroid gland, thyroidectomy, vocal cord paralysis, thyroid surgery, perioperative complications

## Abstract

**Background/Objectives**: This study aims to assess the rate of complications in patients who underwent thyroid surgery and were monitored post-operatively to explore potential correlations between various parameters that may aid in clinical decision making. **Methods**: An observational retrospective study was conducted on patients who underwent thyroid surgery and were followed up in our Endocrinology Unit. Patients were selected based on strict criteria. The following data were collected: sex; age; type of thyroid disease; pre-operative symptoms due to thyroid pathology; surgical procedures; post-operative complications; histopathological diagnosis; and post-operative blood levels of TSH, PTH, vitamin D, and calcium. **Results**: Among 340 patients, 25.29% had benign thyroid disease. Total thyroidectomy was performed in 89.4% of cases. Recurrent laryngeal nerve injury was found in 32 patients. Hypocalcemia occurred in 14 patients within 24 h post-operatively. Histopathological examination identified incidental parathyroid tissue in 5.88% of thyroidectomy specimens. Post-operative hypoparathyroidism was observed in 26 patients, and vitamin D deficiency in 68 patients. **Conclusions**: The study demonstrated that thyroid surgery is quite a safe procedure; however, complications may occur. A statistically significant correlation was found between the type of surgery and the risk of vocal fold palsy, without correlation with the type of thyroid disease. A thorough pre-operative evaluation by a multidisciplinary team may help reduce the risk of post-operative complications. Despite the extensive knowledge of thyroid surgery, small refinements may further improve surgical outcomes.

## 1. Introduction

In recent years, the incidence of thyroid disease has increased considerably. The widespread availability of early screening methods and advanced imaging techniques has enabled timely diagnosis and treatment of thyroid disorders [1]. For most malignant and some benign thyroid diseases, total thyroidectomy is the preferred approach. However, when the disease is well confined to a single lobe, a more conservative approach, such as lobectomy or isthmusectomy, may be performed. Additionally, certain benign diseases, such as small non-symptomatic nodules or autoimmune thyroiditis as well as low-risk microcarcinomas, are often managed with active surveillance and follow-up rather than immediate surgery. The choice between a radical approach and a conservative approach is not always easy: there is a large gray area in which the choice of one approach rather than the other is quite controversial. So, the main indications for total thyroidectomy are size more than 1 cm, local compressive symptoms (e.g., dysphagia, dyspnea, and/or hoarseness), risk of malignancy, regional or distant metastases, previous head and neck radiotherapy, patients older than 45 years old, or first-degree relative with differentiated thyroid carcinoma [2,3]. Some studies suggest that total thyroidectomy reduces the risk of reintervention [4]. However, post-operative complications may still occur, although at a low rate. These complications range from the most frequent ones, such as hypocalcemia, recurrent laryngeal nerve (RLN) palsy, and bleeding, to those that occur rarely, such as chylothorax. Common complications can become permanent, causing a significant impairment in patients’ quality of life [5].

This study aims to assess the rate of complications in a large cohort of patients undergoing thyroid surgery and post-operative monitoring in our Endocrinology Unit. The goal is to identify potential correlations between different factors that may help endocrinologists and surgeons in the decision-making process.

## 2. Materials and Methods

We conducted a retrospective observational study on patients who underwent thyroid surgery between January 2015 and March 2022 in different hospitals and were followed up in the Endocrinology Unit of our University Hospital. The study aims to analyze every complication, with the goal of defining their incidence in our sample, their correlations with sex and age, and to identify possible correlations and cause–effect relationships. The study was based only on preexisting, unique data; therefore, no informed consent was necessary. The study was approved by the ethics committee of the University Hospital (approval n° 02/2022). Inclusion criteria were: (1) men and women, (2) from 18 to 90 years old, (3) undergoing total thyroidectomy or hemithyroidectomy surgery, with or without neck dissection, between January 2015 and March 2022, (4) due to benign or malignant thyroid disease, (5) with at least six months of follow-up. Exclusion criteria were: (1) age under 18 years or over 90 years, (2) suffering from parathyroid pathology, (3) finding of vocal cord pathologies on pre-operative laryngoscopy, (4) patients who developed thyroid complications after the first 30 days post-operative, (5) pre-operative hypocalcemia, (6) patients who quit follow-up, (7) lack of post-operative data.

During the pre-operative study, all patients underwent neck US; flexible laryngoscopy; and blood level tests for Thyroid-Stimulating Hormone (TSH), parathyroid hormone (PTH), calcitonin, and calcium. Furthermore, in the pre-operative stage, TSH receptor antibodies (TSI/TRAbs) and anti-TPO and/or anti-Tg antibodies were also assessed in cases of suspected Graves’ disease and Hashimoto’s thyroiditis, respectively. In cases of nodules with suspicious US features (strong hypo-echogenicity, “taller-than-wide” form, ragged edges, microcalcifications, vascular pattern, extrathyroid extension) [4], the diagnosis was achieved by ultrasound-guided FNA.

The extent of surgery was determined pre-operatively based on ultrasound findings and clinical evaluation. In particular, factors such as the sizes and characteristics of thyroid nodules, the presence of suspected ultrasound signs of malignancy (e.g., irregular margins, microcalcifications, marked hypo-echogenicity), lymph node involvement, and clinical suspicion of autoimmune pathology or local compression were considered. Any intraoperative changes were made only in the presence of unexpected findings, such as macroscopic evidence of local invasion or lymph node involvement not detected by imaging. This strategy was adopted to optimize the surgical approach and minimize the risk of post-operative complications as well as the use of intermittent intraoperative neuromonitoring (i-IONM) in all procedures. All patients diagnosed with thyroid carcinoma underwent central lymph node dissection (level VI) in accordance with surgical oncology guidelines [3]. Prophylactic dissection was performed in cases of medullary thyroid carcinoma and high-risk papillary thyroid carcinoma, while therapeutic dissection was conducted in the presence of clinically or radiologically suspicious lymph nodes.

Within 20 days after surgery, all patients received supplemental calcium therapy. The dosage and mode of administration depended on the individual biochemical parameters and clinical needs. The most used formulations included calcium carbonate (e.g., Calcium Sandoz 1000 mg, Cacit D3 1 sachet daily, IdraCal 1 g daily) and active vitamin D analogs (e.g., Rocaltrol 0.5 µg, 2 tablets per day). In cases of severe hypocalcemia, intravenous calcium supplementation was administered as needed. Permanent hypocalcemia was defined if it lasted for 6 months or more. Moreover, in cases of hypocalcemia, the correct measurement of blood calcium levels was assessed by correcting the calcium for albumin. We considered permanent hypoparathyroidism to exist if the PTH value did not recover within 1 year after surgery or if its level was higher than or equal to 10 pg/mL but the patient still needed correction of symptoms of hypocalcemia. Patients who showed low serum vitamin D levels post-operatively were given vitamin D supplementation, including cholecalciferol or calcitriol, based on post-operative vitamin D levels.

In cases of post-operative dysphonia, patients underwent flexible laryngoscopy to assess the mobility of the vocal cords and to diagnose any paralysis or hypomobility.

### Statistical Analysis

From the database of the Endocrinology Unit, we collected the following data: sex; age; type of thyroid disease; pre-operative symptoms caused by thyroid pathology (compressive symptoms, dysphagia, dysphonia due to neoplastic infiltration of the recurrent nerve); rate of post-operative complications and type of complication (hypocalcemia, hypoparathyroidism, dysphonia and hoarseness, dyspnea, post-operative bleeding); definitive histopathological diagnosis (with data about possible parathyroid removal); post-operative blood dosage of TSH, PTH, vitamin D, and calcium; post-operative thyroid parenchyma residues; surgical procedures; post-operative medical therapy; and possible radiometabolic therapy. These data were collected in an Excel sheet. The values were expressed as numbers, percentages, or averages +/− standard deviation. The complications and their outcomes were studied, evaluating their temporal trend at their onset and after three, six, and twelve months (t0, t3, t6, t12). This allowed us to define complications as temporary or permanent.

The cutoff value used to define hypocalcemia was a total calcium < 8.0 mg/dL or serum calcium < 2.1 mmol/L. Vitamin D status was analyzed as a categorical variable with a cutoff of 30 ng/mL. The number of parathyroid glands removed during surgery was obtained from the histopathological report. Permanent hypoparathyroidism was defined as a post-operative serum parathyroid hormone concentration < 14.0 pg/mL, with a persistent requirement for medication to maintain normocalcemia over 6 months.

Univariate analysis was conducted to evaluate the influence of demographic, pre-operative, post-operative, and pathological factors on post-operative complications as potential effect modifiers. Fisher’s exact test was performed to evaluate possible correlations between two categorical variables. A *p*-value (q) < 0.05 was considered statistically significant.

A cluster analysis was performed to detect possible patient groups at higher risk for post-operative complications. Patients were divided based on key variables, including sex, type of surgery (total thyroidectomy vs. hemithyroidectomy), and the occurrence of major complications (vocal cord paralysis, hypoparathyroidism). This approach allowed us to identify patterns of complication incidence across different demographic and surgical subgroups. SPSS 22.0 software (IBM Corp., Armonk, NY, USA) was used for all analyses.

## 3. Results

Of the 340 patients, 71.18% were female (242 patients) and 28.82% were male (98 patients), with a female-to-male ratio of 3:1. The overall mean age was 52.51 ± 13.46 years (range 19–87). According to the post-operative histopathology reports, 86 patients (25.29%) suffered from benign pathology, while 254 patients (74.71%) received a diagnosis of malignant disease (205 patients had papillary carcinoma, 8 patients had Hurtle cell carcinoma (today classified as follicular carcinoma, oncocytic type) [6], 14 patients had follicular carcinoma, and 15 patients had medullary carcinoma). Of the 86 patients with benign pathology, 82.55% had multinodular goiter. Graves’ disease and Hashimoto’s disease were diagnosed in 5.88% (20) and 2.64% (9) of the patients, respectively. Graves’ disease was found in 10 patients with goiter and in 5 patients with carcinoma. All patients with Hashimoto’s disease had a positive histology for malignant disease. A total of 304 (89.41%) patients underwent total thyroidectomy; hemithyroidectomy was performed in 36 (10.59%) patients; 12 patients who had previously submitted to a hemithyroidectomy needed a second surgery of total thyroidectomy. In 68 patients, a neck dissection was also performed, involving the following lymph node stations: central lymph node dissection (CLND) in 47 (13.82%) patients, bilateral neck dissection in 23 (6.76%) patients, right neck dissection in 19 (5.59%) patients, left neck dissection in 10 (2.94%) patients, lymph node dissection also affected in other districts in 2 (0.59%) patients. RLN injury was found in 32 (9.41%) patients: 17 patients had left paralysis, 13 had right paralysis, and 2 had bilateral paralysis. RLN injury varied depending on thyroid disease: 9 (28.12%) patients had undergone surgery due to benign thyroid disease and 23 (71.88%) due to carcinoma. Among the 9 patients with benign thyroid disease, 6 (66.66%) developed a temporary paralysis. Of the 23 patients with malignant disease, 15 (65.2%) developed a permanent paralysis. Furthermore, we found that RLN injury was more common in patients who had undergone total thyroidectomy (71.88%) than in patients who underwent a hemithyroidectomy (28.12%). Indeed, while most patients (52.17%) undergoing total thyroidectomy developed a temporary vocal fold paralysis (VFP), 77.77% of patients undergoing hemithyroidectomy developed a permanent VFP. The association between the type of thyroidectomy (total vs. hemithyroidectomy) and VFP was found to be statistically significant (*p* < 0.001). Moreover, there was not a statistically significant correlation between the risk of VFP and the histology of thyroid lesion (*p* > 0.001). Furthermore, 24 h after surgery, low serum calcium levels (below 8.0 mg/dL) were found in 14 patients out of the 340 examined (4.12%): only 4 patients of these developed a symptomatic hypocalcemia that required intravenous calcium; the others were treated with oral medications. The symptoms included muscle cramps, perioral tingling, and in some cases, positive Chvostek’s and Trousseau’s signs. No severe cases with seizures, laryngospasm, or cardiac complications were observed in our cohort. Almost all patients received oral calcium replacement therapy for at least the next 30 days. The hypocalcemia rate was higher in the group that underwent total thyroidectomy than the group that underwent hemithyroidectomy. Histopathologic examination identified parathyroid tissue unexpectedly in 5.88% (20 of 340) of the thyroidectomy specimens: in 8 histopathological exams the left parathyroid was found, and in 2 cases the right one was found. Subsequently, we compared the presence of parathyroid glands on the histopathological report and the post-operative hypocalcemia that occurred in 10% (2 of 20) of cases. The rate of incidental parathyroidectomy was found to be higher in the group of patients undergoing total thyroidectomy (85%) and in those with cancer (70%). Furthermore, 26 patients (7.64%) showed post-operative PTH blood values below the cutoff. The rate of temporary and permanent hypoparathyroidism was 50% (13/26) and 50% (13/26), respectively. In particular, 4 of 26 (15.38%) patients developed severe hypocalcemia in association with hypoparathyroidism: in 50% of cases, it was permanent. All patients with post-operative hypoparathyroidism had undergone total thyroidectomy, most frequently due to malignant pathology, with 21 cases out of 26 (80.76%). However, we did not demonstrate a statistically significant correlation between the type of surgery (total vs. hemithyroidectomy) and incidental findings of parathyroid in the histological assessment (*p* > 0.001). Vitamin D status was analyzed as a variable with a 30 ng/mL cutoff: 68 patients (20%) showed a level of vitamin D lower than the cutoff. Hypocalcemia was reported in two patients with post-operative hypovitaminosis. Four patients with post-operative low vitamin D levels developed hypoparathyroidism. Other complications that occurred in the target population were two cases of post-operative hemorrhage that required new surgery and one case of intra-operative hemorrhage; four patients developed dyspnea post-operatively, and one patient reported dysphagia in the post-operative period. No case of chylothorax was reported. The mortality recorded in the post-operative period was 0 (Table 1).

We detected three clusters of patients with the features shown in Table 2; these results show how clusters differ mainly by age, sex, and type of surgery. In addition, complications such as vocal cord paralysis and hypoparathyroidism are concentrated in clusters 0 and 1, suggesting that these factors may be associated. This result highlights that male patients and those undergoing total thyroidectomy may have a higher risk of vocal cord paralysis and hypoparathyroidism. Identifying these risk groups could improve pre-operative planning (e.g., patient selection for total thyroidectomy or pre-operative calcium and vitamin D optimization) and guide post-operative management (e.g., closer monitoring of calcium levels, early intervention for hypocalcemia, and targeted voice rehabilitation strategies for those at risk of recurrent laryngeal nerve injury).

## 4. Discussion

Although thyroid surgery is now a common procedure, it is not without risks, as the complications can significantly impact patients’ quality of life. The incidence of complications varies and is influenced by the surgeon’s experience and skills and the volume of thyroid surgeries performed [7]. In our case series, we observed post-operative complication rates of 15% for total thyroidectomy and 2.64% for thyroid lobectomy. The most frequently encountered complications were hypocalcemia (both transient and persistent), vocal cord palsy (both temporary and permanent), and transient hypoparathyroidism. The most prevalent post-operative finding in our study was dysphonia, occurring in 9.41% of patients. Anatomical variations in the RLN increase the risk of nerve injury during thyroid surgery. Moreover, even in the absence of direct mechanical damage, the nerve’s proximity to the thyroid gland could lead to nerve lesion during thyroid surgery such as ischemia, entrapment, contusion, or irritation of the nerve. Regarding vocal cord palsy, Gunn et al. reported 6% of RLN injury cases in a sample of 11,370 patients. This percentage is lower than our case studies, in which 9.41% were affected by this complication. Most cases occurred after total thyroidectomy (23 out of 32 patients) due to thyroid cancer (23 patients out of 32), confirming the findings of Gunn et al. [8]. The role of IONM in thyroid surgery remains a controversial topic. Although IONM has been widely adopted to reduce the risk of RLN, its effectiveness compared with visual identification of the nerve alone is still uncertain. The meta-analysis by Cirocchi et al. [9] evaluated the use of IONM versus visual identification alone, with no clear evidence for one technique over the other. It is important to note that the certainty of the evidence was classified as very low due to the high risk of bias, significant heterogeneity between studies, and wide confidence intervals. Despite these limitations, in our experience, IONM has been systematically used in all procedures to ensure maximum safety in the preservation of the RLN. Although our data do not allow us to compare the effectiveness of IONM with visual identification alone, the low rate of RLN injury found in our case studies supports the routine use of neuromonitoring as a useful tool in thyroid surgery. However, a prospective comparative study is still needed to assess with greater certainty the impact of IONM on clinical outcomes. Also, Malik et al. highlight how the IONM does not provide a significant decrease in the RLN palsy rate during thyroid surgery; however, it entails a higher expense [10]. In order to reduce the onset of complications, Veyseller et al. suggest two techniques to identify the RLN: (1) superior-to-inferior course, that is, detection of the nerve where it comes into the larynx and then superior pedicle ligation; (2) inferior-to-superior course, that is, detection of the nerve in the tracheoesophageal groove and then inferior pedicle ligation. The study demonstrated that the rate of complications was lower using the superior–inferior approach to detect the RLN during thyroidectomies [11]. In their systematic review, Chen et al. summarized the literature concerning therapies for vocal cord palsy: in cases of unilateral vocal fold palsy after thyroidectomy, injection laryngoplasty, voice training, and neurolysis during the first 12 months or laryngeal reinnervation after 12 months were suggested; for bilateral vocal fold palsy, early laterofixation and laser arytenoidectomy with posterior cordectomy after 12 months were recommended [12].

The second complication in our case studies, in terms of incidence, is hypoparathyroidism, with a prevalence of 7.64%. Hypoparathyroidism can occur after direct injury, incidental excision of the parathyroid glands, devascularization, or obstruction of venous drainage [13]. It can be temporary or permanent, and it most commonly presents with the following symptomatology related to the severity of hypocalcemia and hyperphosphatemia: tingling in the extremities of the limbs and perioral area, numbness, muscle cramps, Chvostek’s and Trousseau’s signs, laryngeal spasms, electrocardiogram (ECG) changes (prolonged QT interval), and neurological symptoms. Based on the systematic review of Melikyan et al., we considered permanent hypoparathyroidism if the PTH value did not recover within 1 year after surgery or if its level was higher or equal to 10 pg/mL but the patient still needed therapy for symptoms of hypocalcemia [13]. We reported an incidence rate of 3.82%, lower than Lončar et al., who found an incidence rate from 7.9% to 22.1% using different definitions of permanent hypoparathyroidism [14]. Our results seem to be similar to those found by Melikyan et al., especially since most cases were observed in patients undergoing thyroidectomy for malignant disease [15]. The incidence of transient hypoparathyroidism was considerably lower (3.82%) than that reported by most of the other authors; 77% of patients with post-operative hypoparathyroidism had a positive histopathological specimen for parathyroid glands; 5.88% were incidental findings. Incidental parathyroidectomy is frequently associated with thyroid surgery. Similar results were found by Özden et al.; in their retrospective study, they reported an incidence of incidental parathyroids of 5.8%, with increased risk in cases of total thyroidectomy [16]. However, Özden et al. did not identify any association between inadvertent parathyroidectomy and post-operative permanent hypocalcemia. In their metanalysis, Wang et al. stated that even the parathyroid autotransplantation does not avoid the risk of hypoparathyroidism [17]. So, as demonstrated by Ponce de León-Ballesteros et al., the identification and preservation of at least three parathyroid glands through meticulous dissection and preservation of their blood supply represents the best solution to reduce the risk of post-operative hypocalcemia and permanent hypoparathyroidism [18]. In their retrospective study, Karadeniz et al. identified the factors that might increase the risk of post-operative hypocalcemia: female sex, age < 28.5 years old, low level of pre-operative serum Ca, and total thyroidectomy. So, this study also confirmed that inadvertent parathyroidectomy is not related to a higher risk of post-operative hypocalcemia [19], as we reported in our case series. In fact, only two of the patients with accidental parathyroidectomy developed symptomatic hypocalcemia. In total, 4.88% showed a symptomatic hypocalcemia, slightly lower than the values reported in the literature [20]. Only 1.17% of our patients developed such severe hypocalcemia as to require intravenous therapy. The current literature reports higher rates than ours. In their study, Kazaure et al. found that only 4.86% of 7366 patients needed calcium intravenous therapy [18]. Breuer et al. reported severe hypocalcemia in 1.5% of 68 adult patients who underwent thyroidectomy [21]. According to Lale et al., post-operative hypocalcemia could be related to the following independent factors: female sex, neck nodes dissection, retrosternal goiter, heavier thyroid gland, malignant thyroid disease, and total thyroidectomy [22]. In their systematic review, Sanabria et al. found that ligation of the inferior thyroid artery increases the risk of temporary and symptomatic hypocalcemia but not the risk of definitive hypocalcemia [23]. According to Stedman et al., PTH and calcium levels at the first post-operative day are good indicators of permanent hypocalcemia: in fact, the need for treatment and the frequency of follow-up is based on these values, reducing the risk of over- and undertreatment [24]. Rubin et al. suggested that the 25(OH)D level also represents a significant predictor of post-operative hypocalcemia after thyroidectomy [25]. For this reason, therapy with calcium and vitamin D in both the pre-operative and post-operative periods could significantly reduce the risk of developing hypocalcemia after surgery.

Hemorrhage with associated laryngeal edema and airway compromise occurs in 0.9–2.1% of thyroid surgeries [26], especially in the immediate post-operative period [27]. Our study shows a similar result, with an incidence of 0.88%. Although bleeding is unpredictable, in their population, Doran et al. found male sex to be the greatest risk [28]. This factor was also confirmed by Liu et al., who identified older age, male sex, Graves’ disease, use of antithrombotic agents (>48 h), total thyroidectomy, neck dissection, and previous thyroid surgery as risk factors for bleeding [29]. Sun et al. demonstrated that malignant pathology is not a risk factor since a similar incidence was found in patients suffering from benign pathology [30]. Neither does drain placement after surgery affect post-operative hematoma formation, as reported by Maroun et al. [31].

In rare cases, a post-operative dysphagia has been reported, but the cause is not clear; so, Galluzzi et al. suggested appropriate education before thyroid surgery [32]. No case of chylothorax was reported in our case study.

The role of CLND in the treatment of papillary thyroid carcinoma has long been debated. While therapeutic dissection (when there is clinical or radiological evidence of lymph node metastases) is universally accepted, prophylactic dissection in patients with clinically negative lymph nodes (cN0) remains controversial. According to the review by Dolidze et al. [33], there are some potential benefits of prophylactic dissection: reduced risk of local recurrence, owing to the removal of occult metastases; improved staging accuracy, allowing for better risk stratification; and potentially influencing the choice of adjuvant therapies (such as radioiodine). However, these benefits must be balanced against the associated risks, including transient or permanent hypoparathyroidism resulting from possible vascular damage or accidental removal of the parathyroid. Indeed, the authors report that the incidence of post-operative hypoparathyroidism is higher in patients undergoing prophylactic CLND than thyroidectomy alone. The second risk is the recurrent nerve injury, although they suggest that the main risk is related to the thyroidectomy itself and not necessarily to the lymph node dissection. In our case series, the CLND was performed in accordance with the guidelines of oncology surgery [3], with a personalized approach based on the individual patient’s risk. Thus, therapeutic dissection was performed in cases of suspected or confirmed metastatic lymph nodes; conversely, prophylactic dissection was reserved for patients with high-risk papillary carcinoma. In our study, 13.82% of patients had a central lymph node dissection (level VI), and most of them had a pre-operative diagnosis of papillary carcinoma. However, we did not find a statistically significant correlation between CLND and the risk of hypocalcemia or permanent hypoparathyroidism. The incidence of post-operative hypocalcemia in our cohort was lower than that reported in the literature. This could be attributed to accurate identification and preservation of the parathyroid during surgery, the use of intraoperative monitoring strategies, and timely administration of calcium vitamin D replacement therapy. Therefore, based on our experience and the current literature, we believe that the indication for CLND should be carefully evaluated. Prophylactic CLND may be useful in high-risk patients; however, it should not be routinely performed in all cases of cN0 papillary thyroid carcinoma. Our results suggest that a selective and personalized approach can help reduce post-operative complications without compromising the cancer control of the disease.

However, this study has some limitations. First, it is a retrospective, multicentric study. Moreover, although each surgeon has at least 10 years of experience in thyroid surgery, some degree of surgeon-dependent variability in complication rates is inevitable.

## 5. Conclusions

The analysis of post-thyroidectomy complications highlights that although these procedures are generally routine and quite safe, they are not without risk. The most common complications, such as VFP and hypoparathyroidism, can significantly impact patients’ quality of life. Notably, our findings indicate a statistically significant correlation between total thyroidectomy and VFP. However, no correlation was found with the type of thyroid pathology. Thus, given these results, careful pre-operative evaluations and multidisciplinary discussions are crucial to reducing post-operative complications (e.g., FVP). Continuous refinement of surgical techniques and intraoperative monitoring strategies may further enhance patient outcomes and safety. As the saying goes, “If you always do what you always did, you will always get what you always got”. In thyroid surgery, continuous refinement of surgical techniques and intraoperative monitoring strategies may further enhance patient care and optimize surgical outcomes.

## 6. Key Messages

1. Although thyroid surgery is a routine and quite safe procedure, complications may occur with significant impact on the quality of life of the patient.
2. There is a statistically significant correlation between the type of surgery (total thyroidectomy) and the risk of vocal fold palsy, but no correlation was found with the type of thyroid pathology.
3. A careful pre-operative evaluation and discussion with a multidisciplinary team could be useful to reduce the risk of post-operative complications.

## Figures and Tables

**Table 1:** Characteristics of included patients.

Characteristics	N (%)
**Sex**	
Male	98 (28.82)
Female	242 (71.18)
**Age**	
Mean ± SD	52.51 ± 13.46
Range (years old)	19–87
**Thyroid surgery**	
Total thyroidectomy	304 (89.41)
Hemithyroidectomy	36 (10.59)
Hemithyroidectomy → total thyroidectomy *	12 (33.33)
**Neck dissection**	
VI level	10 (2.94)
Left	19 (5.59)
Right	23 (6.76)
Bilateral	47 (13.82)
Other	2 (0.59)
**Histological diagnosis**	
*Benign*	
Multinodular goiter	74
Follicular adenoma	11
Colloid hemorrhagic cystic nodule	1
Basedow–Graves’ disease	20
*Malignant*	
Papillary carcinoma	205
Follicular carcinoma	14
NIFTP	12
Medullary carcinoma	15
Hurtle cell carcinoma **	8
**Parathyroids in histological specimen**	20 (5.88)
Total thyroidectomy	17 (5)
Hemithyroidectomy	3 (0.88)
Benign thyroid disease	6 (1.76)
Malignant thyroid disease	14 (4.12)
**Post-operative complications**	
***Hypoparathyroidism***	***26 (7.65)***
Permanent	13 (50)
Temporary	13 (50)
***Hypocalcemia***	***14 (4.12)***
***Bleeding***	***3 (0.88)***
***Vocal cord palsy***	***32 (9.41)***
Left	17 (53.13)
Right	13 (40.62)
Bilateral	2 (6.25)
**Total**	**340 (100)**
Total thyroidectomy	23 (71.88)
Hemithyroidectomy	9 (28.12)
Benign thyroid disease	9 (28.12)
Malignant thyroid disease	23 (71.88)
Permanent	18 (56.25)
Total thyroidectomy	11 (61.11)
Hemithyroidectomy	7 (38.89)
Benign thyroid disease	3 (16.67)
Malignant thyroid disease	15 (83.33)
Temporary	14 (43.75)
***Tracheostomy***	***0 (0)***

SD: standard deviation; NIFTP: noninvasive follicular thyroid neoplasm with papillary-like nuclear features. * Cases of hemithyroidectomy that needed a second surgery of total thyroidectomy; ** Follicular carcinoma, oncocytic type according to the WHO 2022 classification.

**Table 2:** Clusters of patients.

Features	Cluster 0	Cluster 1	Cluster 2
**Mean age (years)**	51.1	55.5	58
**Sex**	Female (100%)	Male (100%)	Female (100%)
**Type of surgery**	Total thyroidectomy (90%)	Total thyroidectomy (87.8%)	Hemithyroidectomy (100%)
**Vocal cord palsy**	100% of patients	100% of patients	0% of patients
**Hypoparathyroidism**	100% of patients	100% of patients	0% of patients

## Data Availability

The original contributions presented in this study are included in the article. The data presented in this study are available on request from the corresponding author due to privacy and ethical reasons.

## Abbreviations

The following abbreviations are used in this manuscript:

US	Ultrasound
FNA	Fine needle aspiration
RLN	Recurrent laryngeal nerve
TSH	Thyroid-Stimulating Hormone
PTH	Parathyroid hormone
i-IONM	Intermittent intraoperative neuromonitoring
VFP	Vocal fold paralysis
